# Breastfeeding Symptom Resolution After Sequential Labial–Lingual Frenectomies: A Case Report

**DOI:** 10.1155/crpe/5545986

**Published:** 2024-12-04

**Authors:** Raymond J. Tseng, Jessica Altemara, Sharon Smart

**Affiliations:** ^1^School of Allied Health, Curtin University, Perth, Western Australia, Australia; ^2^North Carolina Tongue Tie Center, Cary, North Carolina, USA; ^3^Department of Pediatric and Public Health Dentistry, Adams School of Dentistry, University of North Carolina, Chapel Hill, North Carolina, USA

**Keywords:** ankyloglossia, case report, lip tie, tongue tie

## Abstract

**Background:** Breastfeeding is vital for infant nutrition, bonding, and overall health. However, challenges can arise with the presence of tethered oral tissues, commonly known as labial (lip) tie or lingual (tongue) tie, otherwise known as ankyloglossia. This case study explores the differential resolution of breastfeeding symptoms in a one-month-old infant with both labial and lingual ties. It outlines the diagnostic process, surgical interventions, and postoperative care, emphasizing the importance of detailed characterization of symptom resolution associated with a lingual and labial frenectomy.

**Case Presentation:** The one-month-old male presented with 11 symptoms of feeding difficulties. Sequential surgical releases were performed for the labial and lingual ties, with a ranula also addressed. Detailed symptom assessments were conducted at one, two, and 4 weeks postsurgery, revealing differential responses to labial and lingual releases.

**Results:** The results show that 81.8% of symptoms resolved after labial and lingual surgeries, with some symptoms responding specifically to either labial or lingual release. The study suggests that surgical intervention can significantly improve breastfeeding outcomes, including for patients who may not have access to sufficient lactation counseling resources.

**Conclusion:** This single case study provides a valuable starting point for the exploration of which dysfunctional breastfeeding symptoms are associated with a labial tie versus lingual tie. Further research with larger samples is warranted to explore optimal treatment strategies for diverse parent–baby dyads experiencing breastfeeding difficulties, or whose access to lactation support services is limited.

## 1. Introduction

Breastfeeding is a fundamental and natural process crucial for infant nutrition, bonding, and overall health [1, 2]. However, the presence of abnormally restrictive oral frenulum connecting the lip or tongue to the underlying oral mucosa can pose challenges for breastfeeding infants [3]. These challenges manifest in various symptoms, including difficulties with latching, ineffective milk transfer, milk leakage, aerophagia, and maternal nipple pain [4–6]. Moreover, infants with ankyloglossia may also face limitations in bottle feeding, further underscoring the multifaceted impact of oral restrictions on infant feeding practices [7].

Recognizing the complexity of these challenges, interdisciplinary breastfeeding management teams have increasingly acknowledged the significance of diagnosing and managing conditions related to ankyloglossia to optimize breastfeeding outcomes [8]. Frenectomy, a surgical procedure, is a viable intervention aiming to enhance the mobility of the tongue and upper lip, thereby addressing the functional limitations associated with ankyloglossia [9]. The success of frenectomy in resolving breastfeeding challenges can vary among infants. In clinical practice, it often involves simultaneous releases of all restrictions to comprehensively address functional limitations. While there is ample support for the surgical release of a restrictive lingual frenum, evidence supporting the release of labial restrictions is limited [10].

The objective of this case study is to contribute to the current literature by providing a comprehensive account of the postsurgical resolution of breastfeeding symptoms. Specifically, this study delves into the specific symptom resolution details in an infant with tethered oral tissues, focusing on the sequential, as opposed to simultaneous, release of restrictive labial and then lingual frenulum. The primary aim is to underscore the nuanced differences in symptom resolution associated with the lip tie, or the tongue tie, or both the lip and tongue ties.

Verbal and written consent was obtained for use of all clinical data, photographs, and clinical notes in this case study. The caregiver has read and approved the case report. IRB approval was obtained in October 2023 (Sterling IRB #11376, Sandy Springs, Georgia) and the CARE checklist used in the preparation of this manuscript.

## 2. Case Presentation

### 2.1. Case History and Assessment

This case study utilizes a detailed history, observational assessments, and a sequential approach to surgical interventions. The single infant was a six-week-old healthy Caucasian male born via uncomplicated vaginal birth at 39 weeks gestation and who presented with a chief complaint of breastfeeding difficulties. Previous lactation support was provided in the hospital at birth by an international board–certified lactation consultant (IBCLC). He had no known medical condition at the time of examination. His birth weight was 3.68 kg, and the weight at time of initial evaluation was 4.28 kg. Seeking further evaluation and intervention, the family consulted a pediatric dentist at an integrative tongue-tie center. The complaints of breastfeeding difficulty and poor weight gain underscored the need for a timely and thorough assessment.

During the initial assessment, a comprehensive case history was collected, including pregnancy, birth, and feeding history. The chief complaint was that the infant was born with a cyst, later determined to be a ranula, under the tongue. Clinical examination also included a review of medical history and parental report of breastfeeding function and symptoms, which identified 11 symptoms indicative of breastfeeding dysfunction (Table 1). The patient's mother stated these symptoms had been relatively stable and had not shown signs of improvement.

**Figure 1:**
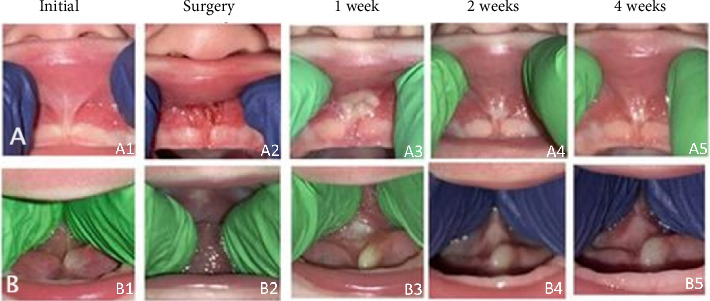
Photographs of healing of the labial (A) and lingual frenulum (B) from initial-assessment (initial), after surgery (surgery), and one-week, two-weeks, and four-weeks postoperative. *Note:* The 2-week postoperative lingual frenulum (B4) photograph does not show the lingual surgical site due to limited patient compliance. Regrowth of the ranula can be noted following lingual release.

Assessment of labial and lingual appearance was completed. The lingual frenulum and labial frenulum were photographed and assessed for signs of restriction via digital exam (Figure 1). The labial frenum was classified as a Class 4 labial frenum with the insertion on the palate, just past the crest of the alveolus [11]. The labial frenum was considered to have sufficient flexibility if the lip could be pushed up to occlude the nostrils with no tension or blanching of tissues in the manipulated area. The patient's lip could not occlude the nostrils, and blanching was noted near the crest of the alveolus (Figure 1A1). Tethering of the maxillary labial frenulum indicated by limited nasal reach has been postulated to cause improper latching to the mother's breast [12, 13]. Thus, coupled with symptoms, this was considered indicative of a restrictive frenulum.

The lingual frenum was classified as a Class 3 lingual frenum with anterior and posterior restrictions, poor flexibility, and mobility [14]. Palpation of the lingual frenum revealed significant anterior restriction such that visualization of the posterior frenulum was poor, partly due to positioning of the ranula. Flexibility and mobility of the tongue were assessed by folding the tongue back and lifting on the posterior segment to see if the tongue tip and blade could touch the highest point on the palate. The tongue tip and blade could contact the palate without significant blanching of the lingual frenum (Figure 1B1).

The infant presented with a visible, fluctuant mass contained within the left half of the floor of the mouth (Figure 1B). This was diagnosed as a ranula based on appearance and medical history. A full assessment of the lingual frenum and the extent of restrictiveness could not be obtained until the ranula had been addressed by otolaryngology. After thorough evaluation of all factors, the three conditions diagnosed were labial and lingual ties, and a ranula on the floor of the mouth.

### 2.2. Management

The Australian Dental Association Policy Statement on Ankyloglossia and Oral Frena highlights that surgical management should be considered after failure of nonsurgical management [15]. Since the infant had accessed previous lactation support from an IBCLC and presented with restricted labial and lingual frenula, based on a thorough case history and functional assessment, surgical management was determined to be the next step in the infant's treatment.

The proximity of the ranula to the lingual frenum dictated a strategic sequence of surgical interventions. The initial step involved the surgery of the restrictive lip tie, with a standard one-, two-, and four-week follow-up schedule. Following this, attention was directed toward the ranula, necessitating care from an otolaryngologist. After ranula correction, persistent symptoms led to the diagnosis and treatment of a tongue tie, with corresponding follow-up visits outlined in Table 1.

### 2.3. Surgical Intervention—Labial and Lingual Frenectomy

The patient was swaddled and positioned on a soft knee-to-knee pad on a dental chair. A CO_2_ laser [16] was used for frenectomy surgeries. The average power was achieved with a contra-angle handpiece and appropriate rate of movement to allow for the desired depth of incision without puncturing muscle or accessory vasculature. Laser settings included 2 W, super pulse mode, 30 msec, 60 mJ, 1200 mW, repeat 20 Hz F1-6.

To facilitate the initial incision, both the lip and tongue were gently retracted with dry gauze ensuring tautness of the frenum. Digital pressure gauged the range of motion following successive laser passes. A cotton swab moistened with distilled water alternated with laser applications for debridement and surgical site visualization. For the lip, the initial incision occurred at the crest of the alveolus, progressing until the lip could be elevated from the base to fully occlude the nostrils without any blanching of tissues (Figure 1A2). Meeting this criterion indicated a complete functional release of the labial frenum. For the tongue, the initial incision was positioned midway between the superior and inferior frenal attachment points. Subsequent incisions were directed toward the tongue's base, ensuring (1) visibility of the genioglossus muscle without restrictive fascia, (2) easy digital pressure of the middle/posterior of the tongue, (3) elevation of the tongue tip to the palate, and (4) no elevation of the mouth floor tissues. Fulfilling these criteria confirmed a complete functional release of the lingual frenum.

### 2.4. Follow-Up Appointments

The infant attended in-person follow-up visits at one, two, and four weeks postsurgical release of the lip tie accompanied by the caregiver. Each assessment included a thorough clinical examination, clinical photography, and a detailed report of current symptoms. Caregiver-rated assessment gauged symptom progression, characterized as “worse,” “same,” “better,” or “resolved” compared to the previous visit. A symptom was considered resolved if it did not occur with any regularity or frequency. Digital palpation was used to monitor flexibility changes following frenum release (Figure 1) to document that the lip could occlude the nostrils without blanching any tissues and that the anterior and mid-tongue could touch the roof of the mouth with no lifting of the floor of the mouth. Criteria satisfaction indicated a successful release and optimal wound healing.

### 2.5. Postoperative Care

Optimal postoperative healing involved maintaining an open wound conformation with vertical healing to prevent frenum reattachment. Postoperative active wound management included exercises, implemented by the caregiver, to ensure optimal healing and to retain full range of motion following release. Digital palpation was applied at the base of the lip until occlusion of nostrils and tongue base until palatal contact was achieved. This process comprised three repetitions of 3 seconds each, repeated every four to 6 hours for 2 weeks then transitioned to four sets of the same, during the waking period, with intervals chosen by the parent. The mother exclusively performed these stretches to ensure consistent and effective postoperative care.

### 2.6. Medical Management of Ranula

A ranula located on the floor of the mouth has the potential to dysregulate normal tongue movement thereby altering breastfeeding ability. The ranula was marsupialized between the two- and four-week follow-up appointments after lip release. The mother reported no significant changes in breastfeeding symptoms or severity, before or after ranula surgery.

### 2.7. After Labial Frenectomy

One lactation support session with an IBCLC occurred one day post–labial frenectomy, addressing basic function assessment and breastfeeding management. A 4-week postsurgical follow-up visit is a commonly utilized timepoint that allows for healing of gingival tissues by both primary and secondary intention resulting in a covering over the surgical site comprised of an intact epidermis, and measurement of breastfeeding improvements after the full four-week stretch regimen has been completed. Specifically, there are several goals that can only be completed at the 4-week follow-up, including completion of our standard 4 week stretch regimen. Other studies looking at wound healing or breastfeeding have recommended 1-week, 2-week, 4-week/1-month, and 6-month follow-up visits [17–19]. During the 4-week visit, the lip exhibited full flexibility, occluding nostrils without frenum blanching (Figure 1A5), a notable improvement from presurgery (Figure 1A1). Specific symptom resolution at the 4-week postoperative visit is outlined in Table 1. This includes the following:

• Full resolution of two symptoms within 1 week sustained throughout the study.
• Partial resolution of five symptoms by the 4-week follow-up, where the right breast symptoms resolved but not the left. No assessment for unilateral differences in breast anatomy or infant muscle tone was performed.
• Leakage during breastfeeding fully resolved for the nose but persisted for the mouth at the lingual frenum consultation.
• No change of four symptoms by the 4-week lip release follow-up.

The parents' overall assessment at the 4-week post–labial frenectomy visit acknowledged general improvement but highlighted the need to address ongoing symptoms for sustainable breastfeeding. With a full range of labial motion confirmed, a decision was made to proceed with a lingual frenum release.

### 2.8. After Lingual Frenectomy

Postresolution of the ranula, the tongue displayed restrictions, including an inability to touch the palate, blanching of the lingual frenulum on elevation, lifting of the floor of the mouth, and no contact between the tongue's most superior position and the palate. By the 4-week postoperative visit, significant improvements were noted, including the tongue touching the palate, no blanching, and no evident lifting of the mouth floor. Despite these improvements, nine symptoms persisted. Specific resolution details at the 4-week postoperative visit for the lingual frenectomy included the following:

• Full resolution of three symptoms that had not previously improved
• Full resolution of four symptoms that had previously improved
• Significant improvement of two symptoms to “occasional” frequency

At the initial consultation, the patient presented with a total of 11 symptoms and a weight of 4.28 kg. Ultimately, the patient resolved 81.8% (9/11) of symptoms and weighed 6.45 kg after the 58-day study period. Differential symptom resolution displayed: labial frenectomy addressed two symptoms, lingual frenectomy addressed three symptoms, with partial resolution of one symptom, and the combination of both labial and lingual frenectomy collectively resolved four symptoms while improving one (Table 1). Interestingly, resolution patterns varied between breasts and between nose and mouth leakage. The parent's overall assessment by the 4-week follow-up emphasized significant improvement in breastfeeding comfort and efficiency, deeming it “so much better than it had ever been before,” and more sustainable than ever before.

## 3. Discussion

This case offers an analysis of symptom resolution after sequential lip and tongue frenectomy to develop a starting point for studies that aim to determine which dysfunctional breastfeeding symptoms (and subsequent resolution) are associated with a lip tie correction, a tongue tie correction, or both a lip and tongue tie correction. The findings in this study enrich the existing literature by presenting a nuanced exploration of symptom resolution following specific surgical interventions, namely, a labial tie division with a follow-up and washout period, followed by a lingual division with the same washout period.

Timeliness of assessment and intervention is important to consider in cases involving breastfeeding infants, particularly if the newborn has faltering growth [20]. Poor weight gain in breastfeeding infants with oral restrictions can result in reversion to bottle-feeding in infants within the first week of life [21]. This case report demonstrated that early consultation and surgical intervention facilitated significant resolution of breastfeeding symptoms, ultimately resulting in sustained breastfeeding. This case report provides documentation of the detailed and differential resolution of dysfunctional breastfeeding symptoms following sequential (nonsimultaneous) labial and lingual tie surgery. The existing literature often lacks specificity regarding symptom resolution related to lip or tongue frenulum. This case addresses this gap, offering valuable insights into the differential effects of releasing lip and/tongue restrictions on breastfeeding outcomes. Baxter et al. [10] reported that over 70% of release providers revised lip and tongue tie simultaneously. Studies on labial frenectomy alone lack specific symptom resolution details [22, 23]. A previous case study by Wiessenger and Miller [24] documented partial improvement in breastfeeding after a lingual frenectomy, followed by full restoration of breastfeeding function after an additional labial frenectomy, which is consistent with the current findings, although specific symptoms were not delineated. In contrast, this case report emphasizes that specific breastfeeding symptoms may resolve after surgery of the lip only, tongue only, or both lip and tongue.

Several breastfeeding symptoms showed complex, multifactorial etiology. For instance, full symptom resolution on the right breast occurred after lip release, while the left breast saw resolution only after tongue release, indicating that structural or anatomical factors may contribute to breastfeeding difficulties. One example is the resolution of a weak or unsustained latch. Although the mother had two interactions with an IBCLC, she did not indicate whether breast and nipple morphology had been comprehensively assessed. In addition, assessment for other peripheral structural issues, such as torticollis, was not conducted. Visits with the surgeon focused on the healing and flexibility of the infant's lip and tongue and focused on the mother's perception of the latch and mechanics at the breast resulting in differential resolution of feeding problems between the left and right breast. From an infant anatomical perspective, the literature offers mixed conclusions on whether the lip, or tongue, or both are the reason for a poor latch. A symptomatic tongue tie is thought to be responsible for difficulties with establishing a latch [19, 25]; however, other studies demonstrate that labial frenectomy only resulted in improved latching ability and generally increased maternal satisfaction and ease of breastfeeding [23, 25]. While it is common to release the tongue first, in this case, the labial frenectomy was completed first due to the presence and subsequent management of a sublingual ranula. Following surgical release, the latch on the right breast immediately improved after labial frenectomy, but the left breast resolved only after both labial and lingual frenectomy. Prior to release, the inclusion of care from an infant feeding expert (IBCLC) ensured that simple breastfeeding management alone would not resolve symptoms. The anatomy of both breasts, variables in the rate of milk flow, ability to create and sustain an effective latch as well as transfer milk effectively, sustainability of feeding patterns, and infant oral structures are important factors in determining optimal sequence and timing of ankylofrenula surgical procedures. Interdisciplinary follow-up support is also recommended to restore breastfeeding function. Further research is needed to explore various breastfeeding symptoms and delineate recovery of labial and lingual tissues, and the factors involved in resolution of each and the interplay.

The combined outcome of sequential lip and tongue frenectomy resolved 81.8% of symptoms, showcasing the significant impact of surgical release on breastfeeding function with just one IBCLC appointment post release for lactation support. It is possible that more involved clinical lactation care would have provided even further resolution of symptoms. However, this report suggests that surgical release with minimal lactation support can still have significant impacts on breastfeeding function as the mother found breastfeeding sustainable with the existing regimen of care.

This study's limitations include a small sample size (one infant) with a relatively mild symptom profile, and a potentially confounding pathological condition of ranula on the floor of mouth, although surgical intervention seemingly had no short- or mid-term impact on breastfeeding symptoms according to the parental report. In addition, improvements were seen following the labial frenectomy after the infant received lactation support, and these positive effects could also be attributed to the breastfeeding intervention. The specific impact of the ranula in breastfeeding dysfunction and the impact of various other factors, such as breast anatomy, infant muscle tone, parental education, external support, or milk supply, remain unclear. Further studies should explore additional symptoms, the sequence of surgeries, and the effects of clinical lactation care in a sequential model.

The takeaway from this case report is that restrictive ties on the lip or tongue can independently cause specific breastfeeding difficulties. This case study, involving a one-month-old infant with both labial and lingual ties, demonstrates that surgical interventions—first for the labial tie and then for the lingual tie—resulted in significant symptom resolution. The findings highlight the importance of sequential surgical approaches and detailed symptom assessment to address breastfeeding challenges effectively. Had this infant received timely access to a comprehensive assessment and treatment of the lip and tongue tie earlier in life, it could have potentially avoided unnecessary weight loss. In addition, the study suggests that with even minimal lactation care for families with limited access to these resources, surgical intervention can substantially improve breastfeeding outcomes. It is worth noting that comprehensive lactation support can substantially improve breastfeeding outcomes, and comprehensive lactation support is always recommended for optimal recovery. This case emphasized the need to consider each frenum site uniquely, recognize discreet symptoms separately, and that tailored interventions can yield meaningful outcomes, offering a valuable perspective for clinicians and researchers to ensure optimal feeding during the critical first few weeks of life [2, 6].

## Figures and Tables

**Table 1:** Differential symptom resolution over time from postoperative visits at one, two, and four weeks for the labial frenectomy, and one, two, and four weeks for the lingual frenectomy.

Symptom present at initial (lip) consult (Day 0)	Labial frenectomy postoperative visit			Tongue consult	Lingual frenectomy postoperative visit		
	1 week (Day 7)	2 weeks (Day 14)	25 days (Day 25)	(Day 30)	1 week (Day 37)	2 weeks (Day 44)	4 weeks (Day 58)
*Symptoms that resolved after lip frenectomy only*							
Baby cannot retain a pacifier on their own	Resolved	—	—	—	—	—	—
Crease mark on baby's upper lip after nursing	Resolved	—	—	—	—	—	—
*Symptoms that resolved tongue frenectomy only (no change after lip frenectomy)*							
After breastfeeding, the baby has signs of spit-up/acid reflux, gagging, or vomiting	No change	Improved, but went back to baseline	No change	No change	Improved	Improved	Occasional⁣^∗^
Clicking noises during feeding with and without nipple shield-air intake during feeding, loss of suction while nursing	No change	No change	No change	No change	No change	Improved	Resolved
Nursing feels like baby is drinking or gulping	No change	No change	No change	No change	No change	No change	Resolved
The baby is excessively gassy—has chronic burping/flatulence or hiccups	No change	No change	No change	No change	Improved	Improved	Resolved
*Symptoms that improved after both lip and tongue frenectomy*							
The latch is weak or unsustained							
Rt. breast	Resolved	—	—	—	—	—	—
Lt. breast	Improved	Improved	Improved	Improved, but still present	Improved	Resolved	—
Baby bobs mouth on and off to latch							
Rt. breast	Resolved	—	—	—	—	—	—
Lt. breast	Improved	Improved	Improved	Improved, but still present	Improved	Improved	Occasional⁣^∗^
Prolonged or incomplete feeding (not fully emptying breast)							
Rt. breast	Improved	Resolved	—	—	—	—	—
Lt. breast	Improved	Improved	Improved	Improved, but still present	Improved	Improved	Resolved
Leakage during breastfeeding from the nose or mouth							
Nose	No change	Resolved	—	—	—	—	—
Mouth	No change	Improved	Improved	Improved, but still present	Resolved	—	—
Signs of discomfort-arching back and clenching hands during and after breastfeeding	Improved	Improved	Improved	Improved, but still present	Improved	Improved	Resolved

⁣^∗^Parent reported that symptoms occurred occasionally but not consistently resolved.

## Data Availability

The data that support the findings of this study are available from the corresponding author upon reasonable request.
